# Reply: Two types of fatigue in cancer patients

**DOI:** 10.1038/bjc.2011.529

**Published:** 2012-01-03

**Authors:** S Singer, A Brown

**Affiliations:** 1 Department of Medical Psychology and Medical Sociology, University of Leipzig, Philipp-Rosenthal-Strasse 55, Leipzig 04103, Germany; 2 Cancer Epidemiology Unit, University of Oxford, Oxford, UK

**Sir,**

Giacalone *et al* (2012) present an interesting idea here in response to our paper on prevalence of fatigue in cancer patients (Singer *et al*, 2011). They assume that fatigue in our group of patients presents as two different types, cancer related and chronic, and that these types need different clinical management. Characteristics of chronic fatigue, in contrast to those of cancer-related fatigue, are that problems appear before the cancer diagnosis, and that they persist for a long period of time. Furthermore, certain characteristics like headache or difficulties concentrating are also specific for chronic fatigue. The latter is traditionally denoted as mental fatigue and was ascertained in our study in addition to general fatigue, on which we based our prevalence data.

Although Giacalone's idea is interesting, we doubt that both types can be distinguished meaningfully in cancer trials or clinical routine. For example, even if fatigue symptoms were prevalent *before* the cancer diagnosis, we cannot assume for certain that these symptoms were unrelated to cancer, as the malignancy might have been developing for a long time before the clinical diagnosis was actually made.

Another reason for scepticism is that, contrary to what Giacalone *et al* assume, the pattern of predicting factors of general and mental fatigue is very similar. We previously investigated that issue in a group of 646 cancer survivors (Kuhnt *et al*, 2009) and saw that both general and mental fatigue are related to the same variables. Thus, different ‘types’ of fatigue could not be reliably classified in our sample of cancer survivors.

Of course, this does not prove in any way that no subtypes of fatigue in cancer patients may exist. However, based on the conceptual considerations and previous empirical data, we have some doubts that such subtypes can be differentiated easily.
